# Microvascular bioengineering: a focus on pericytes

**DOI:** 10.1186/s13036-019-0158-3

**Published:** 2019-03-29

**Authors:** Huaning Zhao, John C. Chappell

**Affiliations:** 1 Center for Heart and Reparative Medicine, Fralin Biomedical Research Institute, 2 Riverside Circle, Roanoke, VA 24016 USA; 2 Department of Biomedical Engineering and Mechanics, Virginia Polytechnic State Institute and State University, Blacksburg, VA 24061 USA; 3 Department of Basic Science Education, Virginia Tech Carilion School of Medicine, Roanoke, VA 24016 USA

**Keywords:** Pericytes, Endothelial cells, Capillary, Microfluidics, Computational modeling

## Abstract

Capillaries within the microcirculation are essential for oxygen delivery and nutrient/waste exchange, among other critical functions. Microvascular bioengineering approaches have sought to recapitulate many key features of these capillary networks, with an increasing appreciation for the necessity of incorporating vascular pericytes. Here, we briefly review established and more recent insights into important aspects of pericyte identification and function within the microvasculature. We then consider the importance of including vascular pericytes in various bioengineered microvessel platforms including 3D culturing and microfluidic systems. We also discuss how vascular pericytes are a vital component in the construction of computational models that simulate microcirculation phenomena including angiogenesis, microvascular biomechanics, and kinetics of exchange across the vessel wall. In reviewing these topics, we highlight the notion that incorporating pericytes into microvascular bioengineering applications will increase their utility and accelerate the translation of basic discoveries to clinical solutions for vascular-related pathologies.

## Background

Oxygen, nutrients, and immune cells are among the many critical elements contained in blood that circulates throughout the human vascular system [1]. The interconnected blood vessels comprising this system are therefore essential for sustaining the health and homeostasis of the tissues and organs in which they reside [2]. Arteries carrying oxygenated blood from the heart ramify into smaller diameter arterioles. Vascular smooth muscle cells (vSMCs) wrap around these vessels to distribute blood into even smaller diameter capillaries where oxygen diffusion and nutrient delivery primarily occur [3]. These intricate microvascular networks also facilitate the removal of carbon dioxide and cellular waste from all tissues. These and other byproducts are returned into the systemic circulation for clearance via small-diameter venules that converge into larger veins. Because microvessels (i.e. blood vessels with a diameter of less than 10 μm) are such a vital component of the vascular system [4], significant effort has been made to engineer various platforms to better understand the biology of the microcirculation as well as to develop clinically relevant, vascular-focused therapies.

The field of vascular bioengineering includes a focus on microvessels and generating functional capillary networks [5, 6] but also encompasses advancing biotechnologies to synthesize larger diameter vessels for bypass grafts [7, 8], for example. Thus, to delineate the scope of this review, we will focus on “microvascular bioengineering”, that is, the biology and technological developments relevant to capillary-sized vessels. As discussed above, the microcirculation is fundamental to the metabolic exchange that sustains every tissue of the human body. Microvessels also regulate the movement of fluid and other solutes across the blood vessel wall [9–14]. The endothelial cells that compose the inner surface of all blood vessels are integral in maintaining this barrier function. Endothelial cells form a selective barrier by assembling various types of junctions amongst themselves including adherins junctions via vascular endothelial-cadherin (VE-Cadherin, or Cadherin5) [15–18] and, highly enriched in neural tissues, tight junctions using zona occludins-1 (ZO-1), claudin5, and/or occludin [11]. Microvascular bioengineering approaches often focus on the formation of these junctions as an important read-out for the success of a particular platform. Equally as important for promoting microvessel barrier function are vascular pericytes [11, 12, 19–21], a cell type that remains poorly understood relative to endothelial cells and is only beginning to be considered in microvascular bioengineering applications.

## Pericyte identity

**Fig. 1:**
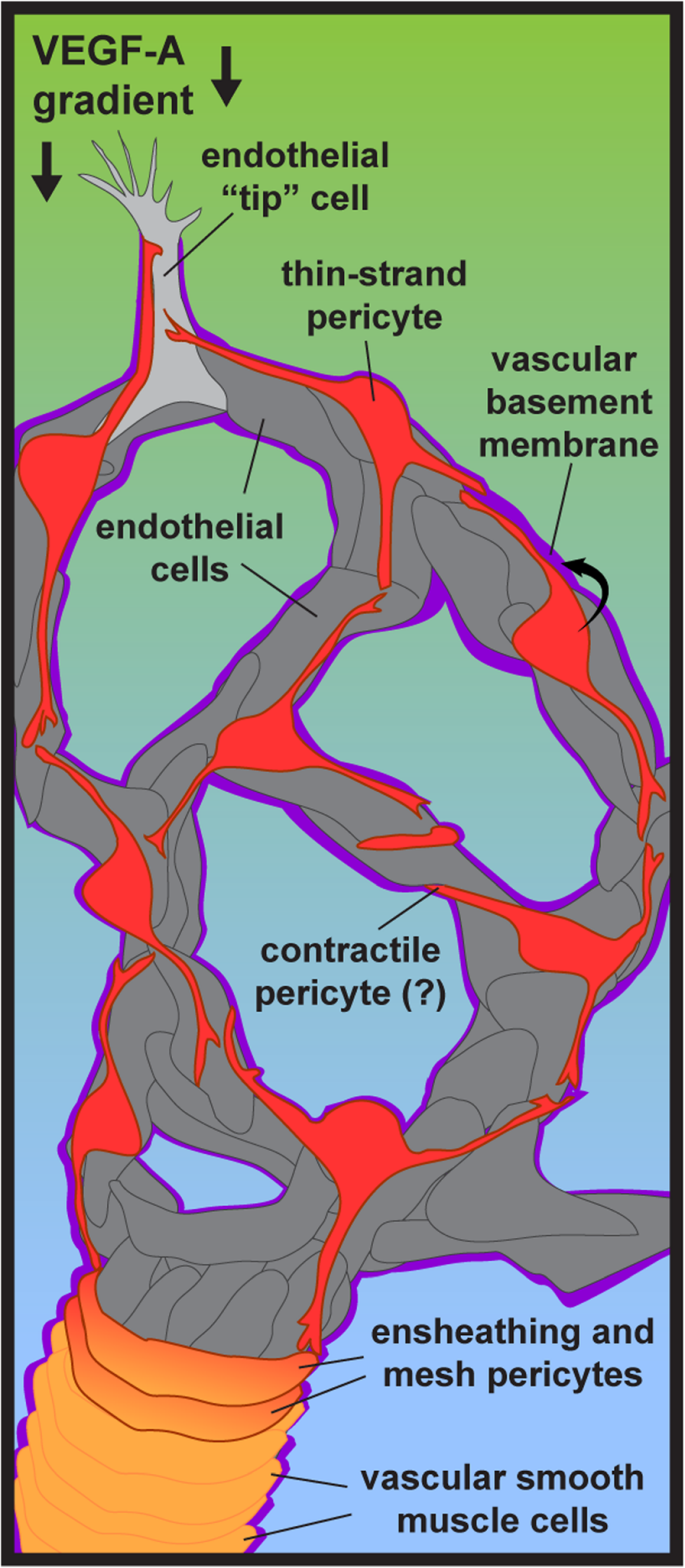
Simplified schematic of pericytes within the microcirculation. During angiogenic remodeling of microvessels, pericytes migrate along endothelial “tip” cells and secrete extracellular matrix components into the vascular basement membrane. Pericytes establish non-overlapping spatial domains, and a subset of pericytes may modulate microvessel diameter. Pericytes closer to terminal arterioles may wrap around and ensheath vessels, though these cells are likely distinct from vascular smooth muscle cells

Pericytes extend along nearly every capillary within the human body, making direct contact with the underlying endothelium and embedded within the vascular basement membrane (vBM). Rouget and Eberth are credited with first distinguishing these cells from vSMCs by noting their unique appearance [22–24]. Specifically, pericytes were identified in perivascular locations but elongated along capillaries, at microvessel branch points and along microvascular segments resembling “bumps-on-a-log” [25] (Fig. 1). Scanning electron microscopy further confirmed their formation of “peg-and-socket” junctions with endothelial cells, and their presence within the vBM, a specialized extracellular matrix (ECM) that surrounds the vascular unit. Vascular pericytes likely arise during embryonic development from unique cellular niches that depend on the specific tissue and organ. Nonetheless, neural crest and primordial mesenchyme are frequently noted as giving rise to pericytes [20, 26–28], with hematopoietic origins also being described (though these may be present primarily during angiogenesis and less so during vessel maturation) [29]. Pericytes depend heavily on platelet-derived growth factor-BB (PDGF-BB) signaling for their recruitment and retention along vessels [30–36]. They highly express PDGF Receptor-β (PDGFRβ), which is a useful cell surface marker for identifying pericytes on capillary branches within many tissues, though interpretation of this signal must also include a consideration that vSMCs and certain brain glia also express PDGFRβ [33, 37, 38]. Neural glial antigen-2 (NG2, gene name: *chondroitin sulfate proteoglycan-4, Cspg4*) is also a helpful marker for pericytes, though oligodendrocyte precursor cells (OPCs) in the brain also express NG2/*Cspg4* [39–41]. Because of this overlap in marker expression, no single marker, or even combination of markers, can be used to specifically identify pericytes. Next-generation sequencing and single-cell profiling techniques will likely yield a more specific marker for pericytes [42–48], but coupling marker expression with morphological features currently offers a high degree of confidence in identifying microvascular pericytes [49].

As criteria for bona fide pericytes continue to strengthen [50], it is becoming possible to identify pericyte subtypes that correspond to their locations within a microvascular network. Grant et al. for example recently conducted a thorough analysis of microvessels in the mouse brain using two-photon and confocal imaging in conjunction with tissue clearing techniques [51]. The authors were able to classify pericytes into three unique categories: 1- ensheathing pericytes (on pre-capillary arterioles), 2- mesh pericytes [on capillaries just downstream of ensheathing pericytes containing α-smooth muscle actin (αSMA)], and 3- thin-strand pericytes (on capillaries immediately adjacent to mesh pericytes) (Fig. 1). Even within these subpopulations, pericytes appear to maintain non-overlapping spatial domains along the microvasculature. These potential distinctions between pericyte subtypes suggest that each subpopulation may play diverse roles within the microcirculation in addition to a subset of common functions [51–54]. Additional studies will be necessary to determine if functional differences do indeed exist among pericyte subtypes, as well as if these pericyte subpopulations are present in other tissues and organs beyond the brain [42]. Complementary imaging approaches will also be useful in characterizing potential pericyte subtypes. Imaging modalities such as super-resolution microscopy, scanning electron microscopy (SEM) [55], and serial block face-SEM [56] can capture ultra-structural details that are likely beyond the resolution of optical microscopes but could be important in understanding the configuration of these pericyte subtypes.

Continued advancement of pericyte-specific tools and markers alongside next-generation imaging and sequencing modalities will not only illuminate distinctions between pericyte subtypes, but will also offer more insight into how pericytes differ from other cell types the reside in perivascular locations. For instance, gross pericyte morphology is well known to contrast with that of vSMCs, which concentrically wrap around endothelial cells in a configuration consistent with their primary role in regulating vascular tone. In spite of these and other distinctions, pericytes have often been grouped together with vSMCs and labeled as “mural cells” [57–59]. Pericytes may indeed give rise to vSMCs as seen in the development of mouse coronary arteries [60]. Distinct molecular signals such as those from the Notch pathway coordinate this pericyte-to-vSMC transition, but these cues do not appear to be required for pericyte differentiation, recruitment, or retention within the microcirculation [37, 61–64]. Pericytes have also been associated with and classified as “perivascular fibroblasts” [58], despite the fact that fibroblasts are rarely, if ever, embedded within the vBM as pericytes are. Additionally, pericytes have been described as perivascular mesenchymal stem cells (MSCs) capable of trans-differentiation and tissue regeneration [53, 65–70], though this identity may be context- and/or model-dependent [67, 71–80]. Therefore, as progress continues in developing markers and tools to identify pericytes and distinguish them from other cell types, microvascular bioengineering approaches will be able to incorporate these essential cells to enhance our understanding of the microcirculation and to generate clinically relevant, microvessel-focused therapies.

## Pericyte function

Pericytes play a variety of important roles in the development, maturation, and functionality of microvascular networks. During the growth of new blood vessel from pre-existing vessels, a process known as angiogenesis, pericytes dynamically interact with endothelial “tip” cells that sprout to form new branches [81–86]. We are just beginning to understand pericyte-endothelial cell crosstalk during sprouting angiogenesis, but what is clear is that pericytes modulate the stability of newly formed microvessel branches [87] and structurally maintain capillary diameter within an appropriate range [88, 89] (Fig. 1). Pericytes also promote and sustain the integrity of the microvessel wall by stimulating endothelial cell junction formation [12–14], as discussed above. In the brain, pericytes appear to provide a level of regulation for the movement of solutes across the vessel wall through transcytosis and vesicular transport [12]. Pericytes also regulate the composition of the blood vessel wall by synthesizing and depositing specific elements within the vBM [20, 88, 90, 91]. Fibronectin, vitronectin, laminins, and Type IV collagen (Col-IV) are among the extracellular matrix (ECM) components that pericytes secrete into the microvessel wall [43, 92] (Fig. 1). In addition to structural regulation of the capillary wall, pericytes have been implicated in dynamic modulation of microvessel tone and diameter, particularly in the central nervous system (CNS) [26, 93–101]. Although pericyte contractility remains an open question [94, 102], recent observations of calcium fluctuations in brain pericytes suggests that they participate at some level in mechanisms coordinating blood flow regulation with region-specific metabolic demand [52]. These diverse roles for pericytes within the microcirculation underscore their importance in maintaining tissue health by promoting and sustaining microvessel stability, barrier function, and perfusion.

Beyond their contribution to vascular-specific functions, pericytes have been implicated in other biological processes, both physiological and pathological. For instance, pericytes have been described as MSCs occupying perivascular locations, suggesting that a subset of vascular pericytes may actually be capable of tissue regeneration [53, 54, 66–68]. A recent study by Guimarães-Camboa and colleagues suggests that we may need to reconsider this role in vivo however, as they found pericytes derived from multiple organs demonstrated lineage plasticity (i.e. pluripotency as MSCs) only when cultured in vitro [80]. Pericyte contributions to certain disease processes have also been reported. In proliferative diabetic retinopathy, “pericyte dropout” (and thus destabilization of the retina capillary wall) is thought to be a key step in the progression of this disorder [103–105]. This loss of pericytes may occur through apoptosis and cell death [106], but may also result from pericyte detachment and emigration away from the microvessel wall [107, 108]. This mode of pericyte loss has been implicated in other disease conditions as well, notably idiopathic pulmonary fibrosis (IPL) [108] and kidney fibrosis [109, 110], among others. In pathologies of fibrosis, pericytes have been identified as major producers of ECM components that exacerbate the fibrotic content of affected tissues and organs [65, 108–112]. An emerging role for pericytes in certain pathological states is their aberrant progression towards a more highly contractile phenotype, as suggested by a notable increase in expression of vasomotor proteins such as αSMA (i.e. hyper-muscularization). In a model of defective col-IV synthesis, for example, pericytes have been shown to acquire higher levels of αSMA [113, 114], as we have also seen in a model of perturbed oxygen sensing [i.e. via mutations in the von Hippel-Lindau (VHL) gene] [115]. Pathological pericyte vasocontractility may adversely affect blood flow within the microcirculation [93, 94, 96, 116] and undermine pressure regulation, which could in turn increase the risk for vessel rupture. Further investigation will be needed to understand the underlying mechanisms that may aberrantly drive pericytes towards a contractile phenotype.

New insights into pericyte biology have emerged with the recent flourish of interest regarding pericyte identity, differentiation, and function within the microcirculation. More detailed understanding of established functions as well as novel roles are still being elucidated, inspiring many thoughtful and comprehensive reviews [14, 19, 20, 25, 117–120]. Indeed, a wide range of intriguing pericyte-focused studies have recently been contributed to the scientific literature; space limitations however prevent an exhaustive review of all of these interesting discoveries. Nevertheless, in this review, we focus on the importance of incorporating pericytes into engineered microvascular constructs across a variety of platforms. We give specific consideration to incorporating pericytes (i) into bioengineered vessels for exploratory purposes and for potential therapeutic applications, and (ii) into computational models of vascular-specific processes.

## Incorporating Pericytes into bioengineered microvessels

The most simplified models of the blood vasculature are often endothelial cell-based, mostly in 2D on cell culture plastic and under static conditions. Human umbilical vein endothelial cells (HUVECs) have served as a predominant source for the cell line utilized, though additional sources have been developed including microvascular endothelial cells. Embryonic stem cells (ESCs) [121–124] as well as induced pluripotent stem cells (iPSCs) [125] have also been used to generate endothelial cells. These basic 2D models were further developed in unique ways to address specific research questions. Endothelial cells and differentiated ESCs and iPSCs have been embedded in 3D matrixes such as Type I Collagen (Col-I) [126, 127] or fibrinogen [128, 129] to investigate vascular remodeling processes such as sprouting angiogenesis and vessel lumen formation [130]. In 2D platforms, endothelial cells have also been subjected to fluid movement and shear stress by a variety of methods [8, 131–135]. These studies in particular ushered in tremendous insight into the coupling between fluid mechanics and endothelial cell biology, including concepts such as endothelial mechano-transduction [136]. Fluidics platforms recapitulating fluid flow across cells in initial configurations were likely more comparable to scenarios of larger diameter vessels. Capillary-like fluidic systems however were relatively limited until the advent of microfluidics technology.

For the models aiming to mimic the microvasculature, incorporation of pericytes represents a logical next step in building complexity and moving closer to modeling in vivo microvessels. Several challenges exist in establishing standard methods to isolate and culture purified pericytes for in vitro use. Because pericyte markers overlap with other cell types, selecting pericyte populations via marker expression (i.e. for magnetic- or fluorescent reporter-based sorting) can yield isolates that are enriched but not necessarily pure. The same obstacles limit validation approaches using certain markers in expression analysis by qRT-PCR and Western Blot, though combinatorial approaches can be useful. An additional challenge in validating pericyte identity in vitro is that their cell fate plasticity may depend heavily on culture conditions [80]. Given that pericyte functions are tightly coupled to endothelial cell activities such as barrier function as well as angiogenic sprouting and remodeling [50, 137], validation strategies that rigorously test for these key features of microvascular pericyte identity, even applied to commercially available cell lines, will bolster confidence in techniques used for their isolation and culture. Similar strategies may also need to be developed for exploring the potential stem cell properties of pericytes, which may exhibit broad plasticity after dissociation from the vessel wall.

Pericyte-endothelial cell co-culture models provide insight into how certain experimental perturbations might affect each cell type directly and perhaps indirectly [57]. Similarly, 3D co-culture [84, 88] or stem cell-based [126, 127, 138] models of vascular remodeling and sprouting angiogenesis capture the unique contributions of both cell types to these processes. Vessel-like structures form within these 3D in vitro models via coalescence of cells into basic vascular networks (i.e. resembling in vivo vasculogenesis such as in the yolk sac [139, 140]). Primitive vasculature in these models can also arise through subsequent endothelial cell sprouting and angiogenic remodeling as observed in vivo in tissues such as the developing mouse retina [115, 141]. Although these 3D in vitro models cannot recapitulate all aspects of the corresponding in vivo scenarios such as including blood flow, oxygen gradients, and the full range of relevant cell types, coupling these models with synthetic or naturally occurring ECM scaffolds [142–144] may also shed light on disease-related phenomena. Pericyte migration away from vessels, as seen in diabetic retinopathy and IPL discussed above, has been successfully modeled with such systems [108].

**Fig. 2:**
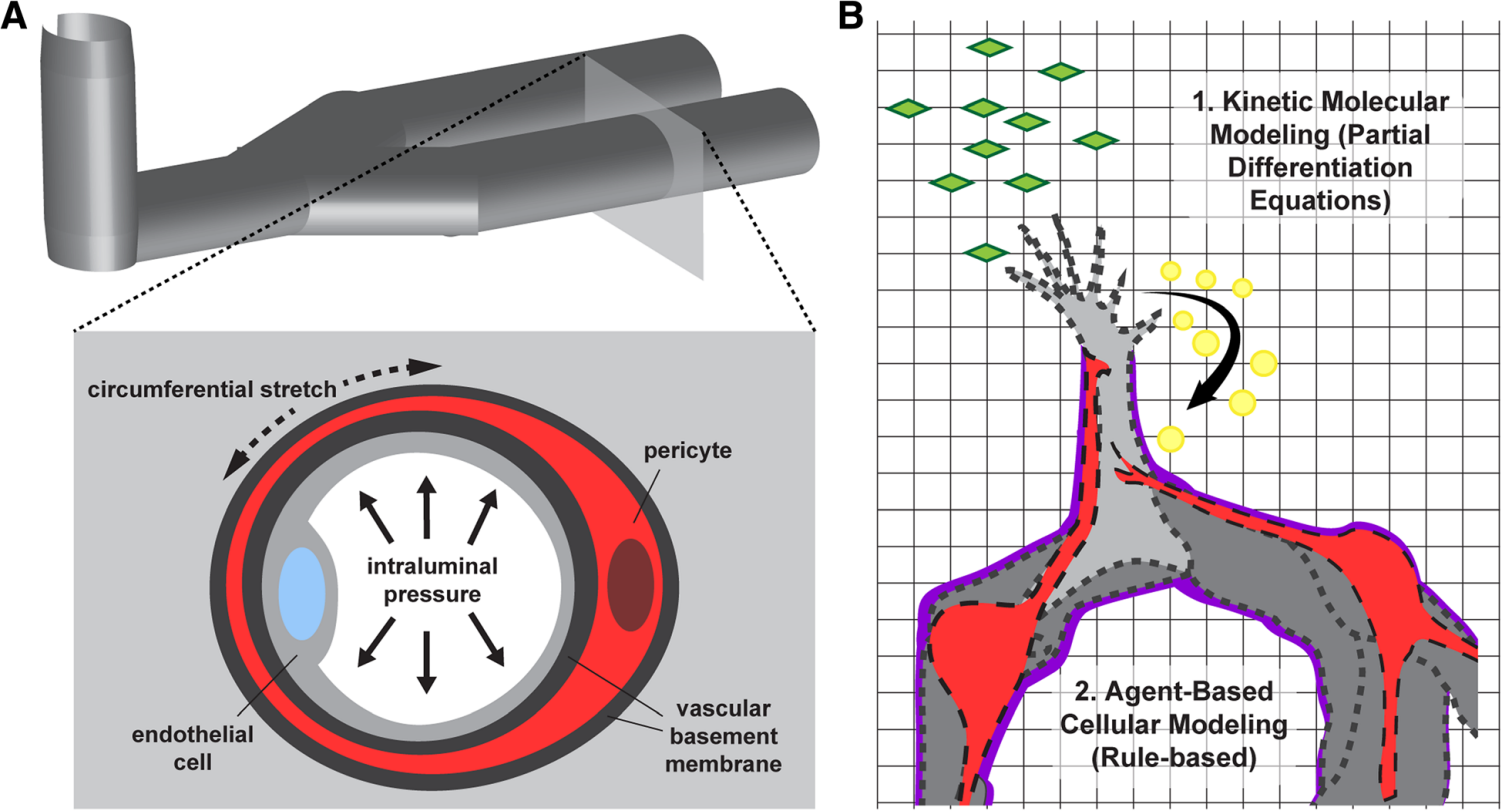
Microvascular bioengineering applications in which pericyte incorporation is relevant. **a** Microfluidic platforms simulating capillaries can address numerous questions regarding pericyte biology including their response to intraluminal pressure dynamics and associated circumferential stresses. **b** Integration of kinetic and agent-based models, such as the one depicted in this simplified illustration, represent how multi-scale computational models might incorporate pericytes to enhance their ability to recapitulate biological processes such as angiogenesis

Incorporating pericytes into vascular fluid mechanics models has been uniquely challenging. A distinct spatial configuration is required for such a platform, that is, positioning endothelial cells only on the “luminal” side while not exposing pericytes to fluid flow on the “abluminal” side. Thus, microfluidic approaches have offered a viable means to create endothelialized micro-channels (i.e. microvessel-like structures) [142, 145, 146] that can be adapted to include pericytes alongside these channels (Fig. 2a). Work from the labs of Steven George and Chris Hughes has yielded one such microfluidics platform that reproducibly develops perfused vessels and allows incorporation of other cell types including pericytes [147–149]. These types of bioengineered microvessels can be further adapted and interrogated to better understand the interface between the microcirculation and cells in the surrounding parenchyma. Blood-brain barrier models for example integrate brain astrocytes (either in basic co-culture and in microfluidics devices) [9] to gain insight into how this uniquely selective barrier is formed and how certain pharmacological agents might transiently disrupt it [10, 150]. Additionally, interactions between surrounding tumor cells and microvessels can be explored in these models to explore effects of chemotherapeutic compounds, efficacy of drug delivery vehicles such as nanoparticles [151, 152], and assessing toxicity profiles for the microvasculature [147]. Bioengineered microvascular fluidics platforms offer tools to better understand the microcirculation during formation of microthrombi and acute pressure changes that might lead to microvessel rupture [153], the risk for which may vary according to pericyte investment. Mechanisms underlying extravasation of leukocytes and perhaps even metastatic tumor cells might also be addressed in these constructs [148]. Pericytes and endothelial cells likely coordinate the selective permeability of the microvessel wall to allow cells to transmigrate from the lumen into the interstitial space [21]. For instance, pericytes may alter their connections with each other and the endothelium, as well as the surrounding vBM, in conjunction with endothelial cell remodeling of their cell-cell junctions [17]. As these microfluidic systems continue to evolve and grow in their utility, so will our insight into the fundamental properties and functions of the microvasculature in sustaining tissue health and in contributing to certain disease conditions [154].

A number of opportunities remain in fully optimizing the design of microfluidic platforms to more faithfully recapitulate the microcirculation. One major challenge is that many vascular channels that form in fluidics devices rarely remain at a diameter size that would be considered on the order of capillaries. Specifically, microvessel diameters typically fall within a range of 3–10 μm, while many microfluidic platforms operate at diameters larger than 10 μm. In addition, the materials used in generating these systems may limit the ability to incorporate multiple cell types along the microvessel wall. These materials may also affect the synthesis and deposition of ECM proteins, causing further divergence from vBM compositions found in vivo. Because the vBM derived from pericytes and endothelial cells provide a level of structural and mechanical integrity to the microvessel wall, the cell-biomaterial interface becomes a very important parameter to optimize for strengthening confidence in the observations made. This challenge is also relevant in use of these models to understand how fluid inside the vessel exerts specific forces on the vessel wall beyond the shear stresses that impinge upon the endothelium. Specifically, intraluminal “blood” pressure creates circumferential, radial, and axial wall stresses that are counteracted by biological elements in vivo (i.e. cell stress fibers, ECM components, etc.) (Fig. 2a). In microfluidic devices, these forces might be absorbed by synthetic elements in the system and may therefore prevent endothelial cells and pericytes from adapting physiologically to their mechanical environment.

Recent technological advances in both biomaterials and micro-fabrication techniques including bio-printing capabilities are pushing microfluidic systems into an era of enormous potential for modeling the microcirculation. These bioengineered models will expand our understanding of microvascular biology and how to use insight into these “first principles” to guide development of clinically relevant therapies for vascular-related pathologies. These rapidly advancing bioengineered microvasculature systems are not without critical limitations however. For example, challenges remain in adequately accounting for key differences and potential heterogeneities in the diffusion barrier of vessels relative to the cell types that may be close spatial proximity to the vessel wall [155]. Work from Dr. Roger Kamm and colleagues for instance demonstrated that biochemical crosstalk with macrophages influences endothelial barrier function and impacts tumor cell migration dynamics, among other modulation of the vessel barrier [155]. Incorporating vascular pericytes into these novel bioengineered microvessel platforms will be an important component of overcoming current technological hurdles, which will usher in new insights into the complex biology of the microcirculation that is relevant to tissue engineering applications [6], cancer immunotherapy [156, 157], and beyond.

## Computational modeling of microvascular Pericytes

As our appreciation of biological complexity grows with each new discovery, we must also develop tools and methods to integrate those insights into working models that will (i) enhance our understanding of biological systems at all levels, and (ii) generate new hypotheses to test, yielding new discoveries and model refinement. Computational modeling represents a primary example of such a tool that facilitates synthesis of data sets and observations from a wide range of experimental systems [158]. In addition, properly validated computational models can offer a means for exploring specific perturbations that might be beyond what is feasibility in experimental models. The field of vascular biology has benefited from the application of in silico models to a variety of questions focused on the microcirculation. Computer simulations have been developed for sprouting angiogenesis [159–161] (Fig. 2b), systemic and localized growth factor kinetics [162, 163], and microvascular biomechanics [164], as well as for oxygen/nutrient exchange within tissue microcirculation [101, 165] and drug delivery across the microvessel wall [166]. Endothelial cells have been the focus for many of these models, but as we learn more about how pericytes influence endothelial cell function (and vice versa), it will be important to build upon previous models and incorporate the pericyte compartment into the parameter space, rule sets, and governing algorithms.

### Computational models of angiogenesis

Angiogenic remodeling is a highly dynamic process involving coordination of numerous cellular behaviors through complex and interconnected signaling networks [161]. Experimental observation of these events yields data sets from various levels including transcriptional, molecular, and cellular. This information guides model construction with regard to rule sets for specific cell activities such as endothelial cell migration or filopodial extensions, as seen in agent-based modeling (ABM) approaches [159, 164, 167]. Multi-scale models couple these actions to underlying molecular pathways and kinetic modeling where each molecular species is accounted for by specific equations [158] (Fig. 2b). This type of modeling has been used to address the complexities of Vascular Endothelial Growth Factor-A (VEGF-A) signaling and crosstalk with the Delta-like 4 (Dll4)-Notch pathway during angiogenic sprouting [16, 168–170], among other signaling mechanisms. Recently, Ubezio et al. utilized complementary experimental and computational models to demonstrate the importance of dynamic fluctuations in endothelial Dll4 levels for normal blood vessel growth [168]. It is becoming increasingly apparent that vascular pericytes are present on, and track very closely along, sprouting endothelial cells [83], suggesting that these and other similar models might provide additional insight into angiogenesis by considering the potential involvement of pericytes.

The various roles that pericytes may play during angiogenesis are still being elucidated. Sprouting endothelial cells are known to secrete PDGF-BB [171], to which pericytes respond by maintaining close proximity to these outwardly migrating cells. Because of their proximate location to endothelial sprouts, pericytes are likely capable of directly or indirectly influencing Notch signals exchanged by endothelial “tip” and “stalk” cells [83]. Pericytes may also provide feedback regulation of angiogenic sprouting by a variety of other mechanisms. For instance, pericytes secrete Angiopoietin-1 (Angpt1) that binds Tie2 on the endothelium to stabilize microvessels, attenuate vascular remodeling and permeability, and induce quiescence [172]. Pericytes have also been proposed to regulate VEGF-A signaling via synthesis of VEGF-A receptors [173, 174], but this role may be context-dependent as observations from a range of models suggest that pericytes produce little, if any, of the known VEGF receptors [43–46, 175–179]. In addition to pro- and anti-angiogenic signaling regulation, pericytes also make unique contributions to the ECM at the pericyte-endothelial cell interface as well as surrounding developing vessels, i.e. the vBM [43, 90]. These ECM components such as fibronectin, collagens and laminins provide structural stability for nascent vessels [126], and are also known to retain and present growth factors that modulate angiogenesis [30, 31, 180]. Our collective understanding of these and emerging modes of pericyte involvement in angiogenesis is still expanding, and as it does, it will be useful to integrate these molecular mechanisms and cellular behaviors into new and existing computational models of angiogenesis to gain even more insight into how endothelial cells and pericytes coordinate the formation of new blood vessels.

### Mathematical approaches to growth factor kinetics

In contrast to the models described above that capture the localized growth factor effects, in silico models have also been developed to describe the systemic distribution and effects of soluble growth factors and their receptors. Kinetics of the VEGF-A pathway for example have been implemented in computational models, giving predications for VEGF-A concentrations in the blood as well as for levels of soluble VEGF-A receptors such as soluble Flt-1 (sFlt-1/sVEGFR1) [162, 163]. These models require a precise accounting of all sources of both VEGF-A ligands as well as VEGF-A receptors. For this reason, studies implicating pericytes as potential sources of VEGF-A ligand and/or its receptors [173, 174] are important to validate and further establish the extent to which these potential pericyte sources of VEGF-A ligands and receptors are functionally relevant [57]. Similar analysis is likely warranted for other signaling pathways related to pericyte function, such as the PDGF-BB pathway. Soluble isoforms of PDGFRβ have been described in several contexts [137, 181] including the developing brain [182], which may be able to diffuse into the systemic circulation and exert effects more broadly. Computational platforms that can simulate both local and systemic PDGF-PDGFRβ dynamics will be essential for understanding how pericytes might respond to both near-field and circulating cues.

### Microvascular biomechanics

Studies exploring the effects of biomechanical cues on the microcirculation have largely focused on shear stresses from blood flowing along the apical surface of the endothelium [8, 131–135]. As mentioned above, intraluminal blood pressure also exerts forces on the microvessel wall, namely circumferential, radial, and axial wall stresses [135]. Pericytes within the vessel wall certainly experience these mechanical inputs and likely respond in specific ways such as contracting [183] or perhaps increasing ECM deposition into the vBM. Confirmation that pericytes contribute to vessel wall compliance in response to intraluminal pressure was provided recently by studies in which pericytes were selectively ablated [19, 184]. In microvessel regions void of pericyte investment, capillary diameters passively increased until a pericyte extension restored coverage in these areas [184]. These observations support the idea that pericytes contribute to the mechanical properties and structural integrity of the microvessel wall, and should therefore be included in computational models focused on capillary biomechanics during both angiogenic remodeling and microvascular homeostasis.

### Microcirculation transport modeling

Models of oxygen diffusion and nutrient exchange create another window into one of the most important functions of the microcirculation [185]. Measuring oxygen diffusion and nutrient/waste exchange in vivo poses numerous technical challenges, some of which are being addressed by recent methodological advances [35, 101, 186]. Complementing the development of experimental approaches, computational methods have been used to predict solute exchange throughout microvascular networks [187]. These approaches are essential to understanding how physiological and pathological changes in the microvessel wall, including in the pericyte compartment and with their associated ECM, can affect distribution of nutrients and oxygen within a tissue. Sweeney et al. recently developed a mathematical model that also captured pericyte contributions to cerebral blood flow regulation by acting primarily as signaling conduits to activate vSMCs upstream [101]. Dynamic imaging of the mouse cortical microvasculature provided corresponding experimental observations in support of this model, demonstrating the utility of combining high-power imaging modalities with rigorous computational methods. Similar approaches focused on drug delivery applications can provide insight into how these vehicles might be designed for optimal transfer within the microcirculation [151]. Given the importance of pericyte contributions to the microvessel wall, and perhaps in regulating endothelial uptake and transcytosis [12], it will be important to integrate pericytes into these models to better predict how certain drugs cross from the bloodstream into surrounding tissues.

## Conclusions

As transcriptional profiling and high-resolution imaging technologies continue to advance at an exciting pace, so too will our understanding of pericytes and their important contributions to the microvasculature. New insights will allow us to effectively incorporate pericytes into in vitro and in silico bioengineered constructs and more faithfully replicate essential features of in vivo microvascular networks. These novel platforms will facilitate testing new therapeutic approaches to enhancing microvascular growth in clinically relevant scenarios. They will also strengthen our ability to screen new and existing drug compounds for intentional and unexpected effects on the microcirculation [188], and specifically on microvascular pericytes [149]. For example, drugs given to myocardial infarction or stroke patients to induce vasodilation and restore tissue perfusion might actually have deleterious effects on pericytes, directly or indirectly (e.g. reperfusion injury), and contribute to “no reflow” within capillary networks [93, 96, 189, 190]. Cell-based therapies harnessing iPSC technology and the like can also be evaluated in these pre-clinical models, accelerating the translation of basic discoveries into medical solutions. It is therefore imperative to continue sharpening our knowledge of pericytes, uncovering their potential as drug targets as well as increasing the fidelity of bioengineered microvascular constructs.

## Abbreviations

ABM: Agent-Based Model
CNS: Central Nervous System
Col-I: Type I Collagen
Col-IV: Type IV Collagen
Dll4: Delta-Like 4
ECM: Extracellular Matrix
ESC: Embryonic Stem Cell
HUVEC: Human Umbilical Vein Endothelial Cell
IPL: Idiopathic Pulmonary Fibrosis
iPSC: Induced Pluripotent Stem Cell
MSC: Mesenchymal Stem Cell
NG2: Neural Glial Antigen-2
OPC: Oligodendrocyte Precursor Cell
PDGF-BB: Platelet-Derived Growth Factor-BB
PDGFRβ: Platelet-Derived Growth Factor Receptor-β
SEM: Scanning Electron Microscopy
vBM: Vascular Basement Membrane
VE-Cadherin: Vascular Endothelial Cadherin
VEGF-A: Vascular Endothelial Growth Factor-A
VEGFR1: Vascular Endothelial Growth Factor Receptor-1
VHL: Von Hippel-Lindau
vSMC: Vascular Smooth Muscle Cell;
ZO-1: Zona Occludins-1
αSMA: α-Smooth Muscle Actin
